# New Therapeutic Perspectives in the Treatment of Uveal Melanoma: A Systematic Review

**DOI:** 10.3390/biomedicines9101311

**Published:** 2021-09-24

**Authors:** Mario Damiano Toro, Lucia Gozzo, Luciano Tracia, Marco Cicciù, Filippo Drago, Claudio Bucolo, Teresio Avitabile, Robert Rejdak, Katarzyna Nowomiejska, Sandrine Zweifel, Yacoub A. Yousef, Rashed Nazzal, Giovanni Luca Romano

**Affiliations:** 1 Department of Ophthalmology, University of Zurich, 8091 Zurich, Switzerland; toro.mario@email.it (M.D.T.); Sandrine.zweifel@usz.ch (S.Z.); 2 Department of General and Pediatric Ophthalmology, Medical University of Lublin, 20079 Lublin, Poland; robertrejdak@yahoo.com (R.R.); katarzynanowomiejska@umlub.pl (K.N.); 3 Department of Biomedical and Biotechnological Sciences, University of Catania, 95123 Catania, Italy; f.drago@unict.it (F.D.); bucocla@unict.it (C.B.); giovanniluca.romano@unict.it (G.L.R.); 4 Clinical Pharmacology Unit, Regional Pharmacovigilance Centre, University Hospital of Catania, 95123 Catania, Italy; 5 Plastic and Reconstructive Surgery Department, American Hospital Dubai, Dubai, United Arab Emirates; ltracia@ahdubai.com; 6 Department of Biomedical and Dental Sciences, Morphological and Functional Images, University of Messina, AOU ‘G. Martino’, 98124 Messina, Italy; mcicciu@unime.it; 7 Centre for Research and Consultancy in HTA and Drug Regulatory Affairs (CERD), University of Catania, 95123 Catania, Italy; 8 Center of Research in Ocular Pharmacology—CERFO, University of Catania, 95123 Catania, Italy; 9 Department of Ophthalmology, University of Catania, 95123 Catania, Italy; t.avitabile@unict.it; 10 Department of Surgery/Ophthalmology, King Hussein Cancer Center, Amman 11941, Jordan; yyousef@khcc.jo; 11 Shami Eye Center, Amman 11941, Jordan; rashednazzal@yahoo.com

**Keywords:** uveal melanoma, treatment strategies, pharmacological treatment, local treatment, systemic treatment

## Abstract

Uveal melanoma (UM) is a rare disease, but the most common primary intraocular cancer, mostly localized in the choroid. Currently, the first-line treatment options for UM are radiation therapy, resection, and enucleation. However, although these treatments could potentially be curative, half of all patients will develop metastatic disease, whose prognosis is still poor. Indeed, effective therapeutic options for patients with advanced or metastatic disease are still lacking. Recently, the development of new treatment modalities with a lower incidence of adverse events, a better disease control rate, and new therapeutic approaches, have merged as new potential and promising therapeutic strategies. Additionally, several clinical trials are ongoing to find new therapeutic options, mainly for those with metastatic disease. Many interventions are still in the preliminary phases of clinical development, being investigated in phase I trial or phase I/II. The success of these trials could be crucial for changing the prognosis of patients with advanced/metastatic UM. In this systematic review, we analyzed all emerging and available literature on the new perspectives in the treatment of UM and patient outcomes; furthermore, their current limitations and more common adverse events are summarized.

## 1. Introduction

Uveal melanoma (UM) is a rare disease (5% of all melanomas), but the most common primary intraocular cancer in adults (mean age of 60) [1,2]. It is more frequent in Caucasians, with an incidence of 0.69 per 100,000 person-year for males and 0.54 per 100,000 person-year for females. In Europe, UM incidence increases with latitude and range from 2/106 in Spain and Italy, 4–6/106 in Central Europe, and >8/106 in Denmark and Norway [1,3].

UM develops most often in individuals with fair skin, light eye color, ocular or oculodermal melanocytosis, cutaneous or iris, or choroidal nevus. Even if it is frequently associated with BAP1 or BRCA1 mutation carriers [1], its pathogenesis is not yet clearly understood [2]. The choroid is the most frequent localization (85–91%), whereas the ciliary body or the iris are affected in only 9–15% of cases [3]. However, iris melanomas is often detected early, resulting in the best prognosis [4], while ciliary body melanomas are associated with the worst prognosis [5]. Half of all patients with UM will develop metastatic disease, whose prognosis is still poor (6–12 months of survival) [6,7].

Based on the conclusions from the Collaborative Ocular Melanoma Study (COMS) [7], globe–vision-preserving radiation therapy is the primary treatment of choice for most UMs nowadays in the developed world [1]. Other globe-preserving therapies may include surgical or laser. In particular, radiation therapy modalities include brachytherapy, photon-based external-beam radiation, and charged-particle radiation [8,9,10,11]. Incidentally, glucocorticoids and anti-VEGF are used for different ocular diseases [12,13,14,15,16,17], including radiation maculopathy secondary to radiotherapy for UM [18,19,20,21].

Plaque brachytherapy involves an affixing dish-shaped source of radiation onto the sclera to cover the base of the intraocular tumor. During radioactive plaque therapy, radiation travels through and is sequentially absorbed by the sclera, the tumor, the retina, the vitreous, and normal ocular structures as it exits the eye [8,9,10,11].

Prior to introduction of plaque therapy, patients with the diagnosis of UM used to be treated by enucleation (surgical removal of the eye globe) as first-line treatment, despite the critical consequences regarding vision and quality of life [22,23,24]. Therefore, the introduction of plaque therapy significantly improved the management of these patients, allowing preservation of the globe, and saving vision in selected cases. For brachytherapy, reported local recurrence rates are 14.7%—for 106Ru treatment, 7%–10% for 125I, and 3.3%—for 103Pd. Brachytherapy does not lead to increased or decreased survival rates as compared to enucleation [3,7,22].

On the other hand, photodynamic laser photocoagulation and trans-pupillary thermal therapy (TTT) reduce local recurrences by the activation of light-sensitive compounds and free radicals, directly focusing energy to the damage tumor. TTT is effective, particularly in cases of limited lesions with few risk factors. To date, no adjuvant chemotherapy allows to prolong survival [3].

Although brachytherapy is the most common globe-preserving treatment option for small- and medium-sized UM patients, the treatment is associated with severe adverse reactions. Indeed, brachytherapy can lead to the onset of radiation-induced retinopathy, cataracts, neovascular glaucoma, and macular edema, with consequent impaired vision within 2 years [3,23,25,26].

Despite the progress in the development of new therapeutic strategies for ocular tumors, to date, all treatments are still unsatisfactory in terms of disease control, as the average treatment failure in all radiation therapies is 6.15%, 18.6% in surgical, and 20.8% in laser therapies [4,27,28]. Thus, the development of new treatment modalities with a lower incidence of adverse events and a better disease control rate are highly demanded. In this regard, new therapeutic approaches emerged in recent decades, highlighting interesting pharmacological targets, such as sigma receptors [29,30].

This systematic review analyzes the data available on the therapeutic perspectives in the treatment of UM and patient outcomes; furthermore, current limitations and more common adverse events are summarized.

## 2. Materials and Methods

### 2.1. Search Methods for Identification of Studies

This systematic review was conducted and reported in accordance with the preferred reporting items for systematic reviews and meta-analyses guidelines [31]. The review protocol was not recorded at study design, and no registration number is available for consultation.

The methodology used for this comprehensive review consisted of a systematic search of all available articles exploring the current available treatments or ongoing trials on UM treatment, localized or metastatic types, in adult subjects.

A comprehensive literature search of all original articles published up to December 2020 was performed in parallel by two authors (L.G. and L.T.) using the PubMed, Cochrane library, Embase, and Scopus databases. For the search strategy, we used the following keywords and Mesh terms: “uveal melanoma”, “pharmacological treatment”, “local treatment”, and “systemic therapy”. Furthermore, the reference lists of all identified articles were examined manually to identify any potential studies that were not captured by the electronic searches.

The same terms have been used to conduct a parallel analysis on clinicaltrial.gov, to identify ongoing clinical trials.

The search workflow was designed in adherence to the preferred reporting items for systematic reviews and meta-analyses (PRISMA) statement [31].

### 2.2. Eligibility Criteria

All studies available in the literature, reporting on original data on UM treatments, localized or metastatic types, were included, without restriction for study design, sample size, and intervention performed. Review articles, protocols, and studies without efficacy data were excluded.

### 2.3. Data Collection

After preparation of the list of all electronic data captured, two reviewers (M.T. and G.L.R.) examined the titles and abstracts independently and identified relevant articles according to the eligibility criteria. Any disagreement was assessed by consensus and a third reviewer (Y.A.Y.) was consulted when necessary. The reference lists of the analyzed articles were also considered as potential sources of information.

The following data were analyzed for each article, using an Excel spreadsheet:

- Study design: retrospective, prospective, comparative and non-comparative, randomized and non-randomized, open, and case report/case series;
- Clinical outcomes: primary and secondary, efficacy, safety, PK;
- Number of eyes studied, number of patients enrolled;
- Primary treatment;
- Follow-up (duration of the study);
- Main results;
- Side effects.

For unpublished data, no effort was made to contact the corresponding authors.

## 3. Results

**Figure 1:**
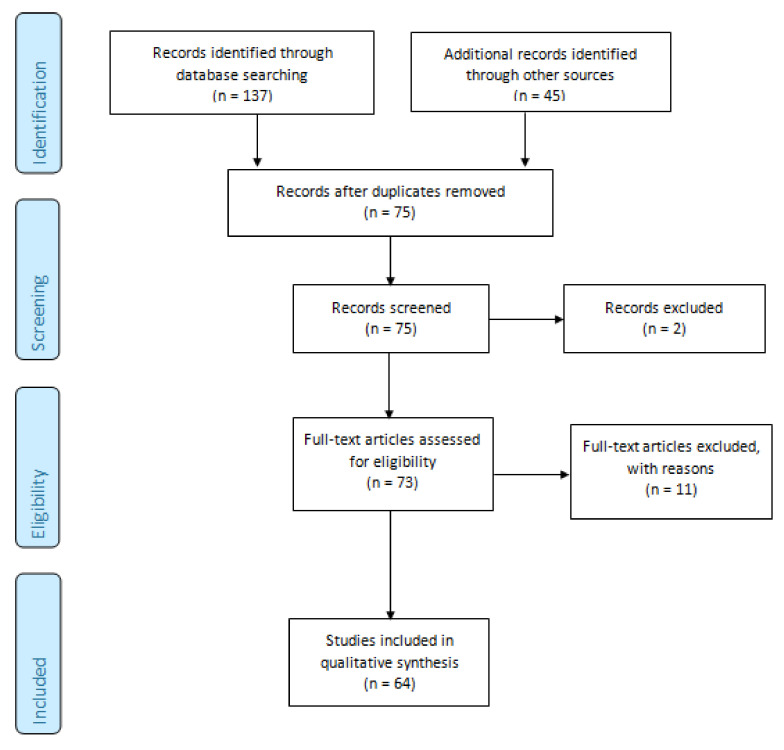
Preferred reporting items for systematic reviews and meta-analyses (PRISMA 2009) flowchart [18]. The 64 studies included in this systematic review were divided in the following groups: treatment of local complications (n = 3; Table 1), local treatment of primary tumor or metastases (n = 13; Table 2), and systemic therapy (n = 48; Table 3). Only one study was a randomized, double blind, placebo-controlled phase III trial; the other studies were phase I/pilot studies (n = 14), phase II studies (n = 34), other designs/design not available (n = 16).

The results of the search strategy are summarized in Figure 1. From 75 articles extracted from the initial research, 73 abstracts were identified for screening and 64 of these met the inclusion/exclusion criteria for full-text review. Moreover, 11 articles were excluded, including 4 studies without efficacy data, 3 protocols, 1 without full-text, 1 non-original article (Figure 1).

Table 4, Table 5 and Table 6 summarize the main efficacy and safety results of these studies.

No data synthesis was possible for the heterogeneity of available data and the design of the available studies (i.e., case reports or case series). Thus, the current systematic review reports a qualitative analysis, detailed issue-by-issue below in narrative fashion.

Among the 13 studies about the local treatment of the primary tumor or metastases from UM, we found 4 clinical trials—3 phase II studies and 1 phase III study. One of these trials assessed the local tumor control of the intravitreal administration of ranibizumab [33], but all patients required enucleation, and the study was terminated early due to the lack of therapeutic advantages.

Olofsson R et al. [23] observed an overall radiological response of 68% in 34 patients with liver metastasis from UM treated with isolated hepatic perfusion (IHP) within a phase II trial. The time to local progression was 7 months and the median overall survival (OS) 24 months, with a significant survival advantage compared to the control group (National Patient Register; *p* = 0.029). All patients enrolled in the phase II study of Fiorentini G et al. [28] obtained an objective response of liver metastasis treated with hepatic transarterial chemoembolization (TACE) adopting irinotecan-loaded microspheres. Finally, the prospective, randomized, phase III trial of Leyvraz S et al. [24] compared the IV or intra-arterial hepatic (I.a.H.) fotemustine administration in 171 patients with liver metastases from UM followed for a median of 5 years. I.a.H. did not improve OS (median 14.6 months) compared to IV administration (median 13.8 months; *p* = 0.59). However, there was a benefit on progression free survival (PFS) for HIA (median PFS of 4.5 versus 3.5 months, respectively; 1-year PFS rate 24% versus 8%), and on response rate (10.5% versus 2.4%).

In regard to systemic therapy, 30 phase II studies and 1 phase III were conducted. Almost half of these studies evaluated the administration of various chemotherapy regimens in advanced or metastatic UM [43,52,54,66,68,69,71,72,74,76,77,78,79].

Overall, we found a low response rate and limited advantage in terms of survival.

The study by Lane AM et al. [62] did not demonstrate an advantage of adjuvant interferon treatment in terms of melanoma-related mortality compared to historical controls in 121 patients with choroidal or ciliary body melanoma during a long-term follow-up (approximately 9 years). However, Binkley E et al. [38] recently reported a survival benefit of adjuvant therapy based on sequential low-dose dacarbazine and interferon-alpha in 33 patients with high-risk UM (5-year median OS of 66% (45–80, median not observed) in treated patients and 37% (19–55, median 54 months) in control).

The trials that assessed the role of immunotherapy in patients with unresectable or metastatic UM showed a low rate of response, a median OS of 1 year with nivolumab and pembrolizumab [35,39], and 6 months with ipilimumab [51]. One patient treated with pembrolizumab reached a complete response (CR, ongoing at 25.5 months), but the drug was stopped after the first dose due to the onset of a severe form of diabetes (grade 4).

Target therapies obtained partial response (PR) or stable disease (SD) as the best objective outcome [36,40,44,48,49,57,58,82]. No difference in PFS or OS was observed versus chemotherapy or compared with the expected patient survival [36,48,50,58,82].

The only randomized, double blind, placebo-controlled phase III trial enrolled 129 patients with metastatic UM to receive either selumetinib or placebo plus dacarbazine [42]. The primary endpoint of PFS advantage was not met (median PFS was 2.8 months in the selumetinib + dacarbazine and 1.8 months in the placebo + dacarbazine group).

Finally, we found more than 140 interventional clinical trials (49 ongoing, 79 completed, 1 suspended, 4 withdrawn, and 9 with unknown status) on uveal melanoma listed on the clinicaltrials.gov database. Among the ongoing trials, 11 foresee the enrollment of patients with local disease and 38 patients with metastatic or unresectable uveal melanoma (Table 7a,b).

## 4. Discussion

To date, no drugs have been specifically approved for the treatment of non-metastatic uveal melanoma.

Pharmacological treatments result ineffective, likely due to the incapacity to reach enough concentration into the tumor area in the eye, as result of the characteristics of the posterior segment and the blood–retinal barrier [96]. It is possible that local delivery at the ocular site will obtain better results, in terms of efficacy and safety. Therefore, researchers are developing new drug delivery systems for uveal melanoma and other ophthalmological diseases, thanks in part to nanotechnology [97,98,99].

However, the possibility of using effective drug delivery still represents a big challenge, and further studies are needed to establish whether this new technology could help in the fight against uveal melanoma [100,101,102,103].

Despite advances in diagnosis and local treatment, the overall survival (OS) of patients with uveal melanoma remains poor because of the progression into metastatic disease. Indeed, up to 50% of cases develop metastasis, especially in the liver, at approximately 5 years after treatment of the primary tumor [104,105,106]. This time is shorter in patients with larger neoplasm, especially in those with a higher grade of malignancy [107,108].

Metastatic disease is particularly difficult to treat; available systemic therapy rarely produces durable responses or significant survival benefits. Actually, the reported median survival after detection of metastatic disease is less than 1 year [109].

Moreover, no adjuvant therapy, which may be more active in treating microscopic metastatic tumor, was shown to reduce the risk of disease spread or survival improvement, and would need further studies [104].

The treatment of metastatic uveal melanoma includes systemic chemotherapy, immunotherapy, and molecular targeted therapy. Moreover, local therapies for liver disease (resection, chemoembolization, immunoembolization, radioembolization, isolated hepatic perfusion, percutaneous hepatic perfusion) are also recommended [110].

The most updated clinical practice guidelines [111,112] recommend the enrollment of patients with metastatic disease in clinical trials, if possible. Otherwise, systemic therapies used to treat cutaneous melanoma can be considered, although no regimens demonstrated improved overall survival in uveal disease.

Chemotherapy regimens for cutaneous melanoma (dacarbazine, temozolomide, cisplatin, paclitaxel, treosulfan, fotemustine) have been used in uveal melanoma although with unsatisfactory results (response rate 0–15%, and no survival benefit) [113,114,115].

Immunotherapy has dramatically improved outcomes for patients with advanced cutaneous melanoma, but this clinical benefit has not been observed in metastatic uveal melanoma, probably due to a low mutational burden and low PD-L1 expression [116,117,118].

The randomized phase III trial that led to the approval of ipilimumab did not include patients with uveal melanoma, and subsequent smaller studies found a low response rate (0–5%) and an OS of less than 10 months [64,119,120,121,122].

Even the phase III CheckMate-067 trial, comparing the concomitant use of nivolumab plus ipilimumab versus the monotherapy alone, excluded uveal melanoma patients [123].

A large series of patients with metastatic uveal melanoma treated with PD-1 and PD-L1 antibodies (pembrolizumab N = 38; nivolumab N = 16; atezolizumab N = 2) showed a partial response rate of 3.6%, a median progression free survival (PFS) of 2.8 months, and an OS of 7.6 months [124].

Recently, the results of a single arm phase II trial demonstrated an overall response rate (ORR) of 18%, a median PFS of 5.5 months, and a median OS of 19.1 months in 33 patients with metastatic uveal melanoma treated with nivolumab plus ipilimumab [125]. Considering these results, the usefulness of immunotherapy in uveal melanoma requires additional investigation and many clinical trials are currently ongoing.

A novel bispecific molecule targeting T-cells (tebentafusp, IMCgp100) showed clinical benefit in patients with metastatic uveal melanoma in phase II, and recently, a phase III study [126].

The mechanism of action consists in the redirection of T cells to target the gp100 protein, highly expressed in melanocytes and melanoma cells. The phase III trial assessed OS as the primary endpoint in 378 naïve patients with metastatic uveal melanoma, randomized 2:1 to receive tebentafusp or the investigator’s choice among dacarbazine, ipilimumab, or pembrolizumab.

OS was statistically significantly improved in patients randomized in the experimental group compared to the control group in the first pre-planned interim analysis (OS hazard ratio of 0.51, and estimated 1-year OS rate of 73% for the study drug versus 58% with the investigator’s choice) [127,128]. These data confirm the positive survival benefit of the phase II clinical trial, and might likely support the use of this drug as a potential new treatment for cancer patients with this highly unmet need. Moreover, the drug was granted the fast-track and orphan drug designation by the FDA for uveal melanoma [129,130] and Promising Innovative Medicine designation under the UK Early Access to Medicines Scheme.

In regard to target therapies, BRAF and KIT inhibitors are not included among treatment options, as uveal melanomas usually lack BRAF and KIT mutations. Conversely, the typical mutations in GNAQ and GNA11 genes lead to constitutive activation of the MAPK and PI3K/Akt pathways and therapies that target downstream effectors, such as MEK, Akt, and protein kinase C (PKC) are under investigation, even with disappointing results so far [113].

For example, selumetinib, a potent and highly selective inhibitor of MEK, associated with dacarbazine, showed no significant improvement in terms of PFS compared to dacarbazine alone (2.8 versus 1.8 months, *p* = 0.32) in the phase III SUMIT trial [55]. Similarly, there was no significant difference in ORR (3.1 versus 0%, *p* = 0.36).

According to the underlying molecular mechanisms, target therapies could probably be improved by combinatory strategies [131].

To date, several clinical trials are ongoing to find new therapeutic options, mainly for those with metastatic disease [110]. Many interventions are still in the preliminary phases of clinical development, being investigated in phase I trial or phase I/II (Table 7).

Additionally, the possibility to exploit a possible ocular pharmaceutical RNA-based treatment against differentially expressed miRNAs in different ocular diseases [132,133], including UM, together with the success of these trials, could be crucial for changing the prognosis of patients with advanced/metastatic UM.

## 5. Conclusions

This systematic review shows the lack of well-designed randomized clinical trials so far and confirms the limited advantages, in terms of response and survival of treatment options for UM. Despite the progress in the development of new effective therapeutic strategies, to date, all treatments for UM are still unsatisfactory and patients have a poor long-term prognosis. The future success of ongoing trials could hopefully change the outcome of patients with advanced/metastatic UM.

## Figures and Tables

**Table 1:** Studies about management of local complications in patients with uveal melanoma treated with radiation-therapy.

N	Author, Year	ID	Study Design	Clinical Outcomes	Eyes	Patients	Primary Treatment	Follow-Up
1	Schefler AC et al., 2020 [32]	NCT02222610	Phase IIb, multicenter, prospective, RCT	ETDRS BCVA change from baseline	40	40 patients with radiation-induced macular edema and a resulting decrease in visual acuity	IVT 0.5 mg ranibizumab monthly (Cohort A), monthly ranibizumab with TRP (Cohort B), or 3 monthly ranibizumab followed by as needed (PRN) injections, and TRP (Cohort C)	2 years
2	Murray TG et al., 2019 [33]	/	Randomized, prospective clinical trial	BCVA and SD OCT central retinal thickness at 1 year	/	39 patients with visually compromising radiation maculopathy confirmed by a decline in BCVA and SD OCT documentation of radiation maculopathy	Aflibercept treatment via 1 of 2 regimens: (1) fixed, every-6-weeks treatment or (2) variable, treat-and-adjust treatment centered around 6 weeks	1 year
3	Horgan N et al., 2009 [34]	NCT00441662	Prospective, RCT.	Development of macular edema; visual acuity	/	163 patients (55 patients randomized to the control group and 108 to the triamcinolone group)	All patients were treated with iodine 125 plaque radiotherapy (8000 cGy to the tumor apex); adjuvant TTT applied to the tumor in most cases. Patients in the triamcinolone group received 3 periocular injections of triamcinolone (at the completion of plaque application, at 4- and 8-month follow-up)	18 months

BCVA = best-corrected visual acuity; IVT = intravitreal; PRN = pro re nata; RCT = randomized clinical, trial; SD OCT = spectral-domain optical coherence tomography; TRP = targeted panretinal photocoagulation; TTP = time to progression; TTT = transpupillary thermotherapy.

**Table 2:** Studies about local treatment of primary tumor or metastases from uveal melanoma.

N	Author, Year	ID	Study Design	Clinical Outcomes	Eyes	Patients	Primary Treatment	Follow-Up
1	Venturini M et al., 2012 [35]	/	/	Eventual complications and lesion devascularization, and tumor response	/	5 patients with UM metastatic to the liver	Transarterial chemoembolization with DEBIRI as a first-line therapy	From 8 to 13 months (mean, 10.6 months) after the first chemoembolization procedure
2	Olofsson R et al., 2014 [36]	NCT01785316	Phase II trial	OS comparison, made using data retrieved from the National Patient Register	/	34 patients with isolated liver metastasis from UM	IHP	2 patients still alive with CR after 23 and 69 months, respectively
3	Leyvraz S et al., 2014 [37]	2004-002245-12 and NCT00110123	Prospective, randomized, phase III trial	OS, RR, PFS, and safety	/	171 patients with liver metastases from UM	IV or I.a.H. fotemustine at 100 mg/m^2^ on days 1, 8, 15 (and 22 in I.a.H. arm only), and after 5-week rest period every 3 weeks as maintenance	5.6 years (range 0.25–6 years)
4	van Iersel LB et al., 2014 [38]	2006-005088-25	Phase I dose-escalation study	Optimal oxaliplatin dose in combination with a fixed melphalan dose. Pharmacokinetic analysis, toxicity, response, and survival	/	11 patients (8 colorectal cancer and 3 UM patients, all with isolated liver metastases)	One hour IHP with escalating doses of oxaliplatin combined with 100 mg melphalan	71 months
5	Yamamoto A et al., 2009 [39]	/	Retrospective evaluation of patients treated with either CE with BCNU (phase II study) or IE with GM-CSF (phase I/IIa study)	Prognostic factors associated with OS and PFS in the liver and extrahepatic organs	/	53 consecutive patients with UM	CE with BCNU or IE with GM-CSF for hepatic metastases	/
6	Huppert PE et al., 2010 [40]	/	Pilot trial	Radiographic response of the liver metastases, time to progression and OS	/	14 patients with hepatic metastases from UM	TACE repeated with a mean interval of 8 weeks (range: 4–24 weeks)	From 5 to 58 months
7	Fiorentini G et al., 2009 [41]	/	Phase II study	Safety, feasibility, and tolerance of TACE adopting irinotecan-loaded microspheres, RR, QoL, and survival	/	10 patients with UM metastatic to the liver	TACE with irinotecan (IRI; 100 mg)	The median follow-up time was 6.5 months (range 4–9 months)
8	Voelter V et al., 2008 [42]	/	/	OS	/	22 patients presenting with nonmetastatic UM at high risk of liver relapse	I.a.h. fotemustine (100 mg/m^2^)	Median follow-up was 4.6 years for the experimental group and 8.5 years for the control group
9	van Iersel LB et al., 2008 [43]	/	/	Systemic and regional toxicity, tumor response, disease-free survival, OS	/	19 patients with isolated unresectable liver metastases from a variety of tumors (13 UM)	IHP using 200 mg melphalan.	Median follow-up was 74 months (range 4–137 months)
10	Noter SL et al., 2004 [44]	/	/	Tumor response, progression and survival, toxicity	/	8 patients with UM hepatic metastases	IHP with 200 mg melphalan	/
11	Egerer G et al., 2001 [45]	/	Single-center experience	Safety and efficacy	/	7 patients with isolated hepatic metastases from UM	Fotemustine 100 mg/m^2^ administered IAH over a 4-h period(1 administration per week for 4 weeks, followed by a 5-week rest period, then every 3 weeks until progression or toxicity)	All patients were followed until death or the last visit to the clinic
12	Hussain RN et al., 2020 [46]	/	Phase II, non-randomized, single center trial	Local tumor control	/	7 patients with primary UM that otherwise required radical surgery because of tumor size.	0.5 mg in 0.05 mL of ranibizumab via six IVT injections over 6 months	/
13	Favilla I et al., 1995 [47]	/	/	Response to treatment	/	36 patients with posterior UM	HPD and the photocytotoxicity of PDT	5 years

CE = chemoembolization; IE = immunoembolization; DEBIRI = drug-eluting beads preloaded with irinotecan; GM-CSF granulocyte-macrophage colony-stimulating factor; HPD = hematoporphyrin derivative; I.a.h. = Intra-arterial hepatic; IHP = isolated hepatic perfusion; IV = intravenous; IVT = intravitreal; ORR = overall response rate; OS = overall survival; PDT = photodynamic therapy; PFS = progression free survival; PD = progressive disease; PR = partial response; RR = response rate; SD = stable disease; T = TACE = Hepatic transarterial chemoembolization; UM = uveal melanoma.

**Table 3:** Studies about systemic therapy of uveal melanoma.

N	Author, Year	ID	Study Design	Clinical Outcomes	Eyes	Patients	Primary Treatment	Follow-Up
1	Nomura M et al., 2020 [48]	/	Phase II prospective, multicenter trial	RR; OS, PFS, disease control rate, and toxicity	/	20 unresectable or metastatic mucosal melanoma	Nivolumab monotherapy	1.6 years (range 1.1–2.8 years)
2	Luke JJ et al., 2020 [49]	NCT01835145	Randomized phase II trial	Improvement of the 4-month PFS4 from 15%; PFS, OS, RR, and safety	/	46 UM that is metastatic or unresectable	Cabozantinib versus Chemotherapy	Median follow-up time of 2.1 years (range 1.9–2.3 years)
3	Piha-Paul SA et al., 2019 [50]	/	Phase I, open-label, dose escalation study	Safety analyses, PK, best response, PFS, and duration of OR	/	72 patients with relapsed, refractory advanced solid tumors (10 with UM)	Mivebresib administered in 28 day-cycles, 3 + 3 dose escalation	/
4	Binkley E et al., 2020 [51]	/	Open label phase II study	MFS rate	/	33 patients with high-risk cytogenetics	Adjuvant therapy (sequential low-dose dacarbazine and interferon-alpha)	5 years
5	Johnson DB et al., 2019 [52]	NCT02359851	Single-arm phase II study	ORR, PFS, OS, response duration, incidence of AEs	/	5 patients with metastatic UM naïve to PD-1–directed agents	Pembrolizumab	Median follow-up 11.1 months
6	Shah S et al., 2018 [53]	/	Phase II trial	RR	/	17 Patients with stage IV UM, and no previous chemotherapy	Ganetespib 200 mg weekly (cohort A) or 150 mg twice a week (cohort B)	/
7	García M et al., 2019 [54]	/	Dose-escalation phase I trial	Tolerability, efficacy, pharmacokinetics	/	12 patients with uveal (6) or cutaneous (6) metastatic melanoma	Oncolytic adenovirus ICOVIR5 administered as a single infusion	/
8	Carvajal RD et al., 2018 [55]	NCT01974752	Randomized, double-blind, placebo-controlled, phase III trial	PFS, ORR, duration of response, change in tumor size at week 6, OS, safety and tolerability, and QoL	/	129 Patients metastatic UM	Selumetinib or matched placebo, plus dacarbazine until disease progression, intolerable toxicity, or another discontinuation criterion	Median follow-up for PFS in the selumetinib plus dacarbazine and placebo plus dacarbazine groups was 2.7 and 1.5 months, respectively
9	Schinzari G et al., 2017 [56]	/	Phase II study	ORR, OS, PFS, and toxicity	/	25 patients with unresectable metastases of UM and BRAF wild type	Cisplatin (80 mg/m^e^, day 1), dacarbazine (250 mg/m^2^/day, days 1–3), vinblastine (2 mg maximum, day 1) every 21 days	/
10	Daud A et al., 2017 [57]	/	Phase II randomized discontinuation trial	ORR, PFS, safety and tolerability	/	77 patients with histologically confirmed melanoma (30% UM)	Cabozantinib treatment during a 12-week lead-in stage. At week 12, patients with evidence of response remained on open-label cabozantinib	/
11	Naing A et al., 2016 [58]	/	Phase I study	Safety and tolerability and MTD and PK properties of AM0010; antitumor activity	/	51 patients (33 patients with CRC, RCC, pancreatic ductal adenocarcinoma, ovarian cancer, prostate cancer, non–small-cell lung cancer, or melanoma were enrolled in the dose-escalation cohorts)	6 dose escalation cohorts from 1 to 40 mg/kg	/
12	Carvajal RD 2014 [59]	NCT01143402	Phase II trial	PFS, OS, RR, and safety/toxicity	/	101 patients with metastatic UM who had not received prior therapy with temozolomide or DTIC	Patients were randomized on a 1:1 ratio to selumetinib 75 mg orally twice daily (n = 50) or chemotherapy (temozolomide 150 mg/m^2^ orally daily for 5 of every 28 days or DTIC 1000 mg/m^2^ intravenously every 21 days; n = 51) until disease progression, death, intolerable toxicity	12 months
13	Adjei AA et al., 2017 [60]	NCT00948467	First in-human, multicenter, open-label, phase I, dose-escalation study	Safety profile and DLTs, MTD, and recommended phase II dose (RP2D) of TAK-733, and PK of TAK-733;antitumor activity.	/	51 patients with advanced solid tumors, 12 with UM	Patients received oral TAK-733 once daily on days 1–21 of 28-day treatment cycles	Blood samples obtained at the following timepoints: pre-dose, and at 0.25, 0.5, 1, 2, 3, 4, 6, 8, 10, and 24 h post-dose on days 1 and 21 of cycle 1; pre-dose on days 8 and 15 of cycle 1; 48, 72, 96, and 120 h post-dose on day 21 of cycle 1; and pre-dose on day 1 of cycle 2. AEs were monitored throughout the trial and for 30 days after the last dose
14	Mouriaux F et al., 2016 [61]	EudraCT: 2010-022527-29	Single-arm phase II trial	Non-progression rate at 24 weeks, PFS, OS, toxicity, QoL	/	32 patients with metastatic UM	400 mg twice a day (800 mg daily) of sorafenib until disease progression or unacceptably severe toxicity or an individual decision was made	24 weeks
15	Shoushtari AN et al., 2016 [62]	NCT01252251	Open-label, single-arm, phase II trial	CBR, PFS, and OS	/	14 patients with metastatic UM	Everolimus 10 mg orally daily plus pasireotide LAR 60 mg intramuscularly (IM) once every 28 days until progression or unacceptable toxicity	Median time on treatment was 8 weeks (range: 1–23 weeks)
16	Joshua AM et al., 2015 [63]	/	Phase II, multicenter study	PFS at 6 months, ORR, DOR, DCR, and OS	/	11 patients with metastatic UM	15 mg/kg tremelimumab, administered on day 1 of every 90-day cycle for up to 4 cycles or until progression or intolerance of toxicity	The median follow-up was 11 months (range 2–36 months)
17	Zimmer L et al., 2015 [64]	EudraCT-Number: 2010-021946-22	Multicenter, open-label, phase II study	OS rate at 12 months	/	53 patients with metastatic UM	Ipilimumab was administered intravenously over 90 min at a dose of 3 mg/kg every 3 weeks for a total of 4 infusions	/
18	Lee CK et al., 2015 [65]	NCT02223884	Phase II, open-label, single-arm study	ORR, DCR, PFS, OS, and safety	/	30 malignant melanoma patients who failed chemotherapy containing dacarbazine were enrolled (10 UM)	Intravenous docetaxel (35 mg/m^2^ on days 1 and 8 of each cycle) and carboplatin (area under the curve 3 on days 1 and 8 of each cycle) administered every 21 days	The median follow-up duration was 19.8 months.
19	Dickson MA et al., 2015 [66]	NCT00451880	Phase I study	Safety and tolerability of once daily and twice daily oral administration of XL281 and the MTD; PK and pharmacodynamic effects	/	160 patients with solid tumors were enrolled in the study (6 UM)	XL281 administered orally daily for 28 days (1 cycle). In the absence of progression) or unacceptable toxicity, patients continued on treatment	/
20	Homsi J et al., 2010 [67]	/	Phase II open-label study	Safety and efficacy	/	22 patients	Docosahexaenoic acid (DHA)-paclitaxel (500 mg/m^2^/week) was administered by IV infusion for 5 consecutive weeks in a 6-weeks cycle	/
21	Borden EC et al., 2011 [68]	/	Phase I trial	Safety and efficacy	/	21 patients with metastatic melanoma (cutaneous metastatic melanoma n = 17, and UM n = 4)	IFN-b1a 12 × 10^6^ IU/m^2^ subcutaneously daily with dose escalation after 14 days and if no adverse events >grade 2 to 18x10^6^ IU/m^2^, until disease progression or a dose-limiting toxicity	/
22	Danielli R et al., 2012 [69]	/	Multicenter expanded access program (EAP)	Tumor assessment. Safety.	/	13 pretreated patients with metastatic UM	Induction treatment with ipilimumab 10 mg/kg at weeks 1, 4, 7, and 10; maintenance doses in patients with clinical benefit or at physicians’ discretion at week 24	/
23	Tarhini AA et al., 2011 [70]	/	Multicenter phase II, single arm study	Safety and efficacy.	/	41 patients with stage III or IV melanoma and no prior chemotherapy (10 with primary UM)	Aflibercept 4 mg/kg intravenously over at least 1 h on day 1 of each 14-day cycle	/
24	Bhatia S et al., 2012 [71]	NCT00329641	Phase II study	ORR, PFS, and OS.	/	25 patients with stage IV UM who had received 0–1 prior systemic therapy	Up to 6 cycles of carboplatin (AUC = 6) and paclitaxel (225 mg/m^2^) on day 1 + sorafenib (400 mg twice daily), followed by sorafenib monotherapy until disease progression	/
25	Falchook GS et al., 2012 [72]	NCT00687622	Phase I, Dose-escalation Trial	Dose escalation, cohort expansion, and pharmacodynamic evaluation	/	97 melanoma patients, including 81 with cutaneous or unknown primary melanoma and 16 UM	Trametinib doses ranged from 0.125 mg to 4·.0 mg, administered orally once daily	/
26	Ott PA et al., 2013 [73]	/	Phase I/II, open-label, dose-escalation study	Toxicity and tumor response	/	31 previously treated patients with advanced melanoma (six patients with UM)	40, 80, or 160 IU/m^2^ arginine deiminase (ADI)-polyethylene glycol (PEG) 20 i.m. weekly	/
27	Mahipal A et al., 2012 [74]	/	Open-label, single-institution pilot study	Evaluation of response carried out every 8 weeks. OS and PFS	/	20 patients with metastatic UM expressing c-kit, 17 of whom failed previous treatments	Sunitinib malate 37.5 mg daily continuously in 4-week cycles	/
28	Lane AM et al., 2009 [75]	/	Non-randomized trial	Melanoma-related mortality compared to historical controls	/	121 patients with choroidal or ciliary body melanoma	Adjuvant IFN treatment protocol consisted of 3 MIU IFN administered 3 times per week by subcutaneous injection over a 2-year period	Approximately 9 years
29	Hofmann UB et al., 2009 [76]	/	A clinical study using Simon’s two-stage design	ORR, OS, time to progression, and toxicity	/	12 patients with metastatic uveal melanoma	Imatinib mesylate at a dose of 300 mg p.o. b.i.d. (600 mg daily) until progression or intolerable side effects	Patients were followed for at least 2-month intervals.
30	Bedikian AY et al., 2008 [77]	/	Patients enrolled in two PK studies	Safety, tumor response, and survival	/	27 adult patients with malignant melanoma with surgically unresectable stage III or stage IV disease	Vincristine sulfate liposome infusion (VSLI) at a dose of2.0 mg/m^2^ every 2 weeks (one cycle)	/
31	Penel N et al., 2008	2005-003685-41	Non-randomized phase II trial	Non-progression rate at 3 months	/	13 patients.	Imatinib at dose of 400 mg twice per day orally	/
32	Adjei AA et al., 2008 [78]	/	Phase I, open-label, multiple-dose study	Safety, tolerability, PK, and pharmacodynamics of AZD6244	/	57 patients with advanced solid malignancies (35% malignant melanoma)	Doses of 50, 100, 200, and 300 mg bid. MTD (200 mg bid) or 50% of the MTD dose (100 mg bid) to evaluate the dose that provided the best balance of safety/tolerability	For patients carrying RAS and BRAF mutations = median, 3.5 months; range, 1 to 6 months, greater than for those without a mutation (median, 2 months; range, 1 to 4 months)
33	Schmittel A et al., 2006 [79]	/	Randomized phase II trial	Rate of responses and disease stabilizations, toxic effect, PFS and OS	/	48 patients	1000 mg/m^2^ of gemcitabine plus 3500 mg/m^2^ of treosulfan (GeT) or 3500 mg/m^2^ of treosulfan alone (T)	/
34	Richtig E et al., 2006 [80]	/	/	Relapse-free survival, safety	/	39 patients with uveal melanoma	Adjuvant IFN alfa 2b treatment 3 million units 3 times a week subcutaneously for 1 year after therapy of the primary tumor	/
35	O’Neill PA et al., 2006 [81]	/	Prospective single arm phase II study	ORR, toxicity	/	15 previously untreated patients with metastatic UM	Dacarbazine (850 mg/m^2^) as an IV infusion and treosulfan (8 g/m^2^) every 21 days as an outpatient procedure up to a maximum of 6 cycles	/
36	Schmittel A et al., 2005 [82]	/	Two-cohort phase II clinical trial	Safety and efficacy	/	33 patients were treated: 14 in cohort 1 and 19 in cohort 2.	1000 mg/m^2^ of gemcitabine and 2500 or 3000 mg/m^2^ of treosulfan in cohort 1 and 3500 or 4000 mg/m^2^ in cohort 2, on days 1 and 8 every 4 weeks	/
37	Corrie PG et al., 2005 [83]	/	Phase I trial	(1) MTD of gemcitabine combined with a fixed dose of treosulfan (2) safety, toxicity, and efficacy	/	27 advanced melanoma patients were enrolled, of whom 5 (19%) had UM.	Chemotherapy on day 1 of a 21-day cycle. Fixed dose of 5 g m^2^ treosulfan, preceded by escalating doses of gemcitabine	/
38	Schmittel A et al., 2005 [84]	/	Phase II trial	Efficacy and toxicity	/	19 patients with metastatic UM	30 or 40 mg/m^2^ of cisplatin, 1000 mg/m^2^ of gemcitabine, and 3000 mg/m^2^ of treosulfan on days 1 and 8, repeated on day 29 (maximum of 6 cycles)	/
39	Schmidt-Hieber M et al., 2004 [85]	/	Phase II trial	Number of patients achieving an OR or SD, toxicity	/	11 patients with metastatic UM	Bendamustine, at a dose of 120 mg/m^2^ on days 1 and 2, repeated on day 22	/
40	Keilholz U et al., 2004 [86]	/	Phase I trial	Toxicity, clinical response	/	39 patients with advanced malignancies (33 with UM and 6 with other histologies)	Gemcitabine (1 g/m^2^, followed by treosulfan on days 1 and 8). Treosulfan dose ranging from 2.5 to 4 g/m^2^; subsequent cohorts received either 3 or 3.5 g/m^2^ of treosulfan	/
41	Terheyden P et al., 2014 [87]	/	Non-randomized phase II trial	Response to treatment	/	20 patients with metastatic UM, chemo-naïve (8 patients) and pre-treated	Treosulfan 3500 mg/m^2^ followed by gemcitabine 1000 mg/m^2^	/
42	Pföhler C et al., 2003 [88]	/	Case series	RR, PFS and OS, and toxicity	/	14 patients with metastatic UM, 13 previously untreated and one pretreated with chemoimmunotherapy	Treosulfan + gemcitabine in four different dose regimens	/
43	Bedikian AY et al., 2003 [89]	/	Phase II clinical trial	Response to treatment, safety	/	14 patients with uveal melanoma metastatic to the liver	Temozolomide at a starting dose of 75 mg/m^2^ per day for 21 days every 4 weeks	/
44	Kivelä T et al., 2003 [90]	/	Prospective, multicenter, nonrandomized phase II study	Efficacy and tolerability	/	24 patients with metastatic UM	Bleomycin 15 mg, vincristine 1 mg/m^2^, lomustine 80 mg, and dacarbazine 200 mg/m(^2^), given every 4 weeks for a minimum of 2 cycles. IFN alpha-2b at a dose of 3 × 10(6) IU on cycle 1, and continued at 6 × 10(6) IU 3 times per week	/
45	Pyrhönen S et al., 2002 [91]	/	Open, two-center non-randomized phase II trial	Activity of bleomycin, vincristine, lomustine, and dacarbazine (BOLD) chemotherapy with human leukocyte interferon, as well as the PFS and OS	/	22 patients with histologically proven metastatic UM	15 mg of bleomycin, 1 mg/m^2^ vincristine, 200 mg/m^2^ dacarbazine, and 80 mg lomustine every 4 weeks + interferon (3 × 10^6^ IU daily for 6 weeks followed by 6 × 10^6^ IU three times per week)	/
46	Becker JC et al., 2002 [92]	/	Prospective phase II trial	Activity of a combination of chemotherapy with fotemustine followed by immune-modulation with IL-2 and IFN alfa	/	48 patients with metastatic UM	Fotemustine 100 mg m^−2^ IHA or IV, depending on the metastatic sites involved, subcutaneous IL-2 and IFN alpha(2)	/
47	Ellerhorst JA et al., 2002 [93]	/	Phase II trial	RR and toxicity	/	28 patients, including14 with UM	The starting dose (level 0) of 9-NC was 1.5 mg/m^2^/day taken orally for 5 consecutive days of each week	/
48	Mertens WC et al., 1996 [94]	/	Phase II study	Antitumor activity	/	17 patients with advanced malignant melanoma (cutaneous, mucosal or UM)	Indomethacin 50 mg orally every 8 h, and ranitidine 150 mg orally every 12 h, and reviewed after 2 weeks	/

ADRs = adverse drug reactions; AEs = adverse events; AUC = areas under the concentration-time curves; CBR = clinical benefit rate; CR = complete response; DCR = disease control rate; DoR = Duration of Response; DTL = dose-limiting toxicity; IrAEs = Immune-related AEs; MFS = Metastasis-free survival; MTD = maximum tolerated dose; PK = pharmacokinetic; ORR = overall response rate; OS = overall survival; QD = once daily; PFS = progression free survival; PD = progressive disease; PR = partial response; RR = response rate; SAEs = serious adverse events; SD = stable disease; TTP = time to progression; UM = uveal melanoma.

**Table 4:** Main efficacy and safety results (management of local complications).

N	Author, Year	Main Results	Side Effects
1	Schefler AC et al., 2020 [32]	Mean ETDRS BCVA gains over first 48 weeks: 4.0, 1.9, and 0.9 letters for Cohort A, Cohort B, and Cohort C, respectively (statistically significant difference in mean BCVA values among 3 cohorts, *p* < *0*.001; statistically significant difference in the mean change of BCVA from baseline among the 3 cohorts, Cohort A vs. B, *p* < 0.0001; B vs. C, *p* < 0.0001; A vs. C, *p* = 0.008).	No serious ocular AEs. No cases of endophthalmitis or IO inflammation.
2	Murray TG et al., 2019 [33]	42.5% (showed better than 20/50 BCVA, and only 5% showed a BCVA worse than 20/200). No difference was found between a fixed 6-week treatment and a variable treat-and-adjust interval.	No patients demonstrated endophthalmitis or metastatic disease or died during the study.
3	Horgan N et al., 2009 [34]	The triamcinolone group had a significantly lower risk of developing macular edema (hazard estimate, 0.35; 95% confidence interval, 0.11– 0.58; *p* = 0.004). Other factors predictive of development of macular edema were largest tumor base (*p* = 0.001) and tumor thickness (*p* = 0.018). By multivariate analysis, triamcinolone treatment was the most significant factor associated with a lower risk of macular edema (hazard estimate, 0.45; 95% confidence interval, 0.19–0.70; *p* = 0.001). At 18-month follow-up, moderate vision loss occurred significantly less frequently in the triamcinolone group than in the control group (31% vs. 48%; chi-square, 4.25; 1 df; *p* = 0.039).	In 8 patients (7%), raised IOP developed after the 1st or 2nd triamcinolone injection. Elevated IOP occurred in 15% of the triamcinolone group and in 7% of the control group (chi-square, 1.93; 1 df; *p* = 0.165). All cases were controlled with topical treatment. Rates of cataract progression were similar in both groups. No case of globe perforation associated with periocular injection.

AEs = adverse events; BCVA = best-corrected visual acuity; IO= intraocular; IOP = intraocular pressure.

**Table 5:** Main efficacy and safety results (local treatment of primary tumor or metastases from uveal melanoma).

N	Author, Year	Main Results	Side Effects
1	Venturini M et al., 2012 [35]	Tumor responses: 1 CR, 2 PRs, 1 SD, and 1 PD.	Well tolerated in all 5 patients.
2	Olofsson R et al., 2014 [36]	Overall radiological response: 68% of patients (12% CR, 56% PR, 18% SD, and 15% PD); time to local progression: 7 months; 68% of patients developed extrahepatic metastases after a median of 13 months, and the median OS was 24 months. Significant survival advantage of 14 months (*p* = 0.029) when comparing these patients with a control group.	No postoperative mortality was observed. There were 3 major complications: 1 patient developed a systemic inflammatory response syndrome with respiratory insufficiency as well as renal and cardiovascular failure; 1 patient also developed respiratory insufficiency with pneumonia and pleural effusion; 1 patient perforated duodenal ulcer at the fourth postoperative day.
3	Leyvraz S et al., 2014 [37]	HIA did not improve OS (median 14.6 months) when compared with the IV arm (median 13.8 months) [hazard ratio (HR) 1.09; 95% confidence interval (CI) 0.79–1.50, log-rank *p* = 0.59]. However, there was a significant benefit on PFS for HIA with a median of 4.5 versus 3.5 months, respectively (HR 0.62; 95% CI 0.45–0.84, log-rank *p* = 0.002). 1-year PFS rate was 24% in the HIA arm vs. 8% in the IV arm. Better RR in the IAH (10.5%) compared with IV (2.4%).	In the IV arm, the most frequent grade ≥3 toxicity was thrombocytopenia (42.1%) and neutropenia (62.6%), compared with 21.2% and 28.7% in the IAH arm. The main grade ≥3 toxicity related to IAH was catheter complications (12%) and liver toxicity (4.5%).
4	van Iersel LB et al., 2014 [38]	The AUC of oxaliplatin at the MTD of 100 mg oxaliplatin ranged from 11.9 mg/L h to 16.5 mg/L h. All 4 patients treated at the MTD showed progressive disease 3 months after IHP. Only 8 patients were available for response evaluation of which 3 patients showed a PR, with a duration of response of 6.5–11.1 months. Median OS was 18.7 months.	Dose limiting sinusoidal obstruction syndrome (SOS) occurred at 150 mg oxaliplatin.
5	Yamamoto A et al., 2009 [39]	High-dose IE resulted in significantly better OS (20.4 vs. 9.8 months, *p* = 0.005) and systemic PFS (12.4 vs. 4.8 months, *p* = 0.001). Patients who achieved regression of hepatic metastases after embolization lived much longer than did those who did not achieve regression (27.2 vs. 9.9 months, *p* = 0.001). At multivariate analysis, prolonged OS was confirmed for patients who underwent high-dose IE, and patients with regression of hepatic metastases.	/
6	Huppert PE et al., 2010 [40]	No patient showed CR, 8 patients (57%) showed PR, 4 patients (29%) had SD and 2 patients (14%) had PD. Time to progression ranged between 5 and 35 months (median 8.5 months). Median survival of all patients was 11.5 months (3–69 months) following first TACE and 18.5 months (5–75 months) following diagnosis of liver metastases. At the time of data analysis, 10 patients had died and 4 patients were alive. The survival rate was 86% at 6 months, 50% at 12 months, 28% at 18 months, and 14% at 24 months following first TACE.	Symptoms of post-embolization syndrome were seen in all patients. In 2 patients, the application of additional morphine was necessary to overcome right upper quadrant pain. In 1 patient, acute renal insufficiency occurred after the second TACE.
7	Fiorentini G et al., 2009 [41]	All patients had an objective response; 3 patients had a major response with evidence of metastases reduction of 90%, 3 had a reduction of 80% and 4 presented a reduction between 70% and 60%; 8 patients are alive at the time of this analysis.	The most important AE was abdominal pain during the procedure.
8	Voelter V et al., 2008 [42]	Median survival for patients treated with fotemustine was 9 years (95% confidence interval (CI) 2.2–12.7) compared with 7.4 years in the control group (95% CI 5.4–12.7). The corresponding 5-year survival rates were 75 and 56%, respectively (*p* = 0.539). The estimated hazard ratio for death at 5 years was estimated at 0.98, with a 95% CI of 0.38–2.61, *p* = 0.981	5 patients (23%) received only the induction cycle (3–4 infusions), with early treatment cessation owing to hepatic toxicity (n = 4) and/or catheter-related complications (n = 2). The main side effect of the adjuvant treatment was drug-induced hepatitis; 3 patients experienced grade 3 gastric toxicity; 2 patients presented with grade 3 neutropenia and 1 with grade 3 thrombocytopenia.
9	van Iersel LB et al., 2008 [43]	Of the 12 UM patients perfused, 4 (33%) had a PR, 6 (50%) patients had SD, and 2 (17%) patients were immediately progressive. Median DFS was 6.6 months with a median OS of 10.0 months. 50% of other primary tumors showed at least PR.	Reversible grade 3 or 4 hepatotoxicity occurred in 10 (56%) patients, while VOD occurred in 4 (22%) patients.
10	Noter SL et al., 2004 [44]	No CRs were observed. 4/8 patients (50%) showed a PR. 2 patients showed SD and 2 patients showed progression after 3 months of follow-up. The median TTP was 6.7 months (range, 1.7–16.9 months). The median survival was 9.9 months (range, 4.7–34.6 months). The 1-year survival rate was 50% and the 2-year survival rate was 37.5% with 1 patient still in follow-up 15 months after treatment.	1 patient developed grade 3–4 leukopenia. 3 patients experienced grade 3–4 toxicity of 1 or more liver enzymes. Major complications occurred in 3 patients in the week following the perfusion procedure: VOD in 2 patients and lung embolism in 1 patient.
11	Egerer G et al., 2001 [45]	2 patients achieved a PR, 3 had SD, and in 2 patients, the tumor progressed in the liver. Extrahepatic progression to lung was diagnosed in 1 patient. The median survival was 18 months (range, 3–43 months) with a median TTP of 16 months (range, 0 to 43 months) in 6 patients. The median survival from diagnosis of the primary tumor was 54 months (range, 31–122 months); 2 patients survived for more than 2 years, and 2 patients are alive at present with residual liver metastasis, at 3 and at 43 months.	The toxicity of the locoregional chemotherapy was minimal in the 7 patients
12	Hussain RN et al., 2020 [46]	No patients achieved CR or PR at any visit. All required enucleation. The study was terminated early, as alternative treatments were clearly superior for local tumor control.	/
13	Favilla I et al., 1995 [47]	The longest duration of tumor control = 6.5 years. 76% of melanomas were not growing at the end of the first year, 62% at the end of the second year, 38% with no signs of growth at the end of the fifth year. Three patients died during the 5-year period: 1 from melanoma metastases after 2 years, the second from a concurrent malignancy, and the third from heart failure after 1.7 years.	The post-treatment visual function was worse in 26 patients (76%). However, in 24 (67%), the tumor was located posteriorly and involved the macula. No eyes were enucleated because of complications of PDT.

AEs = adverse events; AUC = areas under the concentration-time curves; CE = Chemoembolization; IE = immunoembolization; CP = carboplatinum/paclitaxel; CR = complete response; DCR = disease control rate; DoR = Duration of Response; DTL = dose-limiting toxicity; I.a.h. = Intra-arterial hepatic; IHP = Isolated hepatic perfusion; IrAEs = Immune-related AEs; MFS = Metastasis-free survival; MTD = maximum tolerated dose; ORR = overall response rate; OS = overall survival; QD = once daily; PDT = photodynamic therapy; PFS = progression free survival; PD = progressive disease; PR = partial response; RR = response rate; SAEs = serious adverse events; SD = stable disease; T = treosulfan alone; TEAEs = Treatment Emergent Adverse Events; TACE = Hepatic transarterial chemoembolization; TTP = time to progression; UM = uveal melanoma; VOD = veno-occlusive disease.

**Table 6:** Main efficacy and safety results (systemic therapy for uveal melanoma).

N	Author, Year	Main Results	Side Effects
1	Nomura M et al., 2020 [48]	Best ORR = 23.5% (95% confidence interval (CI) 6.8–49.9%)); median PFS 1.4 months (95% CI 1.2–2.8); median OS 12.0 months (95% CI 3.5 to not reached); 1-year OS 50.0% (95% CI 25.9–70.0%).	Treatment-related AEs were mostly grade 1 (pruritus in particular). Treatment-related AEs of grade 3 occurred in 15% (colitis, anemia, adrenal insufficiency, diarrhea), and no grade 4 or 5 AEs were observed.
2	Luke JJ et al., 2020 [49]	10/31 met the primary endpoint of PFS4 (32.3%) compared to 4/15 randomized to arm 2 (26.7%; *p* = 0.350). No difference in PFS was observed (95% CI: 56–162 days) compared with arm 2 (95% CI: 56–152 days; *p* = 0.964, hazard ratio (HR) 0.99 (95% CI: 0.51–1.86)). The median OS in arm 1 was 191 days (6.4 months; 95% CI: 168–314) versus 218 days (7.3 months; 95% CI: 170-NA days) in arm 2 with no difference (*p* = 0.580, HR = 1.21 (95% CI: 0.62–2.34)). The trial was terminated for futility.	Grade 3–4 AEs were 71.0% and 66.7% in arms 1 and 2,respectively. Common attributable grade 3–4 events included fatigue, increased AST or ALT, and thromboembolic events.
3	Piha-Paul SA et al., 2019 [50]	Of 61 evaluable patients from dose escalation, 26 (43%) had SD and 35 (57%) had PD. Median PFS was 1.8 months (95% confidence interval, 1.8–1.9). All solid tumor patients (N = 84) discontinued mivebresib. Primary reasons for discontinuation were radiologic PD (63%), clinical PD (13%), withdrew consent (8%), AE related to progression (3%), AE not related to progression (3%), lost to follow-up (3%), and other (8%).	Most common TEAE were dysgeusia (49%), thrombocytopenia (48%), fatigue (26%), and nausea (25%). Most common grade 3/4 TEAEs were thrombocytopenia (35%) and anemia (6%). Dose-limiting toxicities included thrombocytopenia, gastrointestinal bleed, hypertension, fatigue, decreased appetite, and aspartate aminotransferase elevation.
4	Binkley E et al., 2020 [51]	5-year and median MFS were 64% (44–78) and 79 months in treated patients and 33% (15–52) and 29 months in observed patients. 5-year median OS was 66% (45–80, median not observed) in treated patients and 37% (19–55, median 54 months) in observed patients.	Grade 1 or 2 fatigue was reported by 33 patients (87%). Grade 1 or 2 elevations in transaminases were observed in 14 patients (37%), and grade 1 or 2 depression, in 5 patients (13%). Grade-3 hematological toxicity in 6 patients. No grade 4 AEs were reported.
5	Johnson DB et al., 2019 [52]	1 patient had a CR (ongoing at 25.5 months), and no PRs were observed (RR 20%). 1 patient experienced prolonged SD (ongoing at 11 months), and another had SD lasting 11 months before experiencing progression (clinical benefit rate 60%). The remaining 2 patients experienced rapid PD. Median PFS was 11.0 months and median OS was not reached (median follow-up, 11.1 months; range, 0.4–25.5 months).	The patient who experienced the CR had fulminant type 1 diabetes (grade 4) that arose after the first dose of pembrolizumab, and stopped treatment. Other toxicities included grade 1 hypothyroidism and rash; 3 patients had no side effects.
6	Shah S et al., 2018 [53]	Response outcomes included 1 PR, 4 SD, 11 PD. Overall RR = 5.9%; DCR = 29.4%. PFS = 1.6 months cohort A and 1.8 months cohort B. OS = 8.5 months cohort A and 4.9 months cohort B.	An overall 31% of AEs were grade 3–4 and were mostly related to gastrointestinal toxicities.
7	García M et al., 2019 [54]	No objective responses were observed. At the lower dose levels, 2 patients had SD, at the highest dose level SD was observed in 5/6 patients. The survival probability was 3.7 times longer for the UM patients. Median survival = 271 days for uveal versus 73 days for cutaneous (hazard ratio 0.15; 95% confidence interval 0.026–0.85).	At dose levels 1a–3a, no relevant toxicity was observed. Acute toxicity was mainly a flu-like syndrome with fever, chills, arthromyalgia, headache, nausea, and vomiting, and diarrhea. The first patient at the level 5a experienced transaminitis grade 3 and grade 3 thrombocytopenia.
8	Carvajal RD et al., 2018 [55]	The primary endpoint of PFS was not met. In the selumetinib + dacarbazine group, there were 82 events (85%) compared with 24 (75%) for placebo + dacarbazine; the HR for PFS was 0.78 (95% CI: 0.48 to 1.27; *p* value = 0.32), with no significant benefit of selumetinib. Median PFS was 2.8 months in the selumetinib + dacarbazine and 1.8 months in the placebo + dacarbazine group.	Incidence of AEs of special interest was more frequent with selumetinib plus dacarbazine; however, these were generally grade 1/2.
9	Schinzari G et al., 2017 [56]	PRs were observed in 5 (20%) patients, SD in 12 (48%); DCR was 68%. Median OS of all the patients was 13 months, median PFS 5.5 months. OS of responding patients was 21 months; OS of patients with disease control was 18 months, significantly longer than survival of progressing patients (7 months, *p* = 0.0003).	5 (20%) patients experienced grade 3–4 toxicity.
10	Daud A et al., 2017 [57]	In the UM cohort, 61% of patients (14/23) had SD at week 12, and no patient had a PR, resulting in an overall DCR of 61%. The median PFS for the 23 patients with UM was 4.8 months (41% PFS rate at 6 months) and median OS was 12.6 months. Most patients with UM stayed on study treatment for 44 months, and 6 patients stayed on treatment for >10 months.	The most common grade 3/4 events were fatigue (14%), hypertension (10%), abdominal pain (8%), hand-foot syndrome (5%), asthenia (5%), back pain (5%), and hypokalemia (5%); 6 patients (8%) discontinued study treatment because of AEs; 1 died from peritonitis due to diverticular perforation (deemed related), and 1 from an unknown cause (deemed unrelated).
11	Naing A et al., 2016 [58]	41 (80.4%) of 51 patients were evaluable. 1 patient with UM (40 mg/kg) had a PR, with histologically confirmed reduction of multiple gastric metastases.	AM0010 was tolerated well, with manageable AEs. Most frequently observed AEs were anemia (51%), fatigue (45%), thrombocytopenia (42%), injection site reactions (36%), and fever (30%). Grade 3 to 4 non-hematopoietic AEs were observed in 15%. Grade 3 to 4 anemia or thrombocytopenia was observed in 18%. Only 1 patient discontinued treatment because of a DLT (recurring anemia). A grade 2 rash was observed in 3 patients, and a grade 3 rash was observed in 1.
12	Carvajal RD 2014 [59]	The median PFS was 7 (95% confidence interval (CI), 4.3–8.4) and 15.9 weeks (95% CI, 8.4–21.1) for chemotherapy (n = 49) and selumetinib (n = 47), respectively. The HR for PFS was 0.46 (95% CI, 0.30–0.71; *p* < 0.001) in favor of selumetinib. The median OS was 9.4 (95% CI, 6.0–11.4) and 10.8 months (95% CI, 7.5–12.9) for chemotherapy and selumetinib, respectively, with a HR of 0.79 (95% CI, 0.46–1.37; *p* = 0.40). Tumor regression was uncommon with chemotherapy, with no responses observed. 49% of patients with selumetinib achieved tumor regression.	TEAEs were observed in 97% patients treated with selumetinib, with the most common being acneiform rash (75%), CPK elevation (60%), fatigue (57%), AST elevation (48%), and ALT elevation (42%). Blurred vision (6%) and other visual changes (7%) were observed. 37% experienced grade 3–4 TEAEs, including CPK elevation (13%), AST elevation (7%), and ALT elevation (6%).
13	Adjei AA et al., 2017 [60]	No DLTs were observed in patients who received TAK-733 in the first 8 dose cohorts (0.2–8.4 mg).Subsequently, 4 patients experienced DLTs in cycle 1. Based on the observed DLTs in cycle 1, the MTD of TAK- 733 was determined to be 16 mg once daily on days 1–21 in 28-day treatment cycles.	All patients experienced at least 1 AE of any grade, and 88% reported drug-related AEs. Grade ≥ 3 AEs were reported in 53%. Overall, 67% experienced rash. 14% experienced ophthalmic AEs (visual impairment, photopsia, blurred vision, photophobia, periorbital, and retinal edema); 27% experienced at least 1 SAE.
14	Mouriaux F et al., 2016 [61]	There were no confirmed objective tumor responses. The estimated 24-week PFS was 31.2% (95% CI: 14.8%–47.6%) and the estimated 24-week OS was 62.5% (95% CI: 45.4%–79.6%). The OS rate among patients who received at least 2 months of treatment did not significantly differ compared with the expected patient survival rate (*p* > 0.05).	281 ADRs were reported, including 20 grade 3 or 4 reported in 10 patients. 12 patients (41.4%) required dose modifications due to toxicity.
15	Shoushtari AN et al., 2016 [62]	23% had SD for at least 16 weeks and were considered to have clinical benefits. Overall, SD was the best objective outcome. Median duration of SD in these patients was 8 weeks (range: 8–16 weeks). 6/13 patients had PD on first assessment. Median PFS from first date of treatment was 16 weeks (range: 7–23 weeks); median OS from first day of study treatment was 11 months (range: 4.5–28.5 months).	All patients experienced at least 1 possibly related AE. The most frequently AEs were metabolic (hyperglycemia, hypertriglyceridemia, hypercholesterolemia), gastrointestinal (diarrhea, oral mucositis), or hematologic (leukopenia, thrombocytopenia, neutropenia). The grade 3 AEs were hyperglycemia (n = 7), oral mucositis (n = 2), diarrhea (n = 1), hypophosphatemia (n = 1), and anemia (n = 1). There were no grade 4 or 5 AEs.
16	Joshua AM et al., 2015 [63]	2 of 11 patients received 4 cycles of treatment, whereas the rest only received 1 cycle. No responses were observed; 10 of the 11 evaluable patients had progressed at 90 days. The median PFS was 2.9 months (95% confidence interval (CI) 2.8–3.0) and the 6-month PFS was 9.1%. The median OS was 12.8 months (95% CI 3.8–19.7).	Grade 3 or 4 rash, nausea, and diarrhea was observed in 1 (9.1%), 2 (18.2%), and 3 (27.3%) patients, respectively; 2 patients experienced SAEs secondary to grade 3 and 4 diarrhea; 2 patients developed hyperthyroidism.
17	Zimmer L et al., 2015 [64]	45 pretreated (85%) and 8 treatment-naïve (15%) patients received at least 1 dose of ipilimumab; 1-year and 2-year OS rates were 22% and 7%, respectively. Median OS was 6.8 months (95% CI 3.7–8.1), median PFS 2.8 months (95% CI 2.5–2.9). The DCR at weeks 12 and 24 was 47% and 21%, respectively; 16 patients had SD (47%), none experienced PR or CR.	TEAEs were observed in 66%, including 36% of grade 3–4 events; 1 drug-related death due to pancytopenia was observed. Most common irAEs were gastrointestinal disorders, skin-related toxic effects, and hepatic disorders. The most frequent grade 3 or 4 irAEs were diarrhea (13%) and colitis (11%).
18	Lee CK et al., 2015 [65]	In the intent-to-treat population (n = 30), the median PFS was 3.7 months, and the median OS was 9.4 months. In the per-protocol population, the median PFS was 4.3 months (22 PFS events among 25 patients), and the median OS was 9.6 months (16 OS events among 25 patients). The ORR was 12% (n = 3; 95% confidence interval (CI), 0% to 24.74%), and DCR was 80.0% (n = 20; 95% CI, 64.32% to 95.68%). UM patients (n = 9) had the best prognosis when treated with docetaxel + carboplatin compared to those with other subtypes (median PFS 7.6 months; OS 9.9 months). All patients with UM had the best response as SD.	Most patients (n = 29, 96.7%) reported at least 1 AE related to treatment, and the total incidence of grade 3 or 4 AE was 66.7%. The most common AE was neutropenia (67.7%), with half of the patients experiencing grade 3 or 4. Non-hematologic toxicity was less common and less severe.
19	Dickson MA et al., 2015 [66]	The MTD was 150 mg. 2 patients had PR (1 papillary thyroid cancer with NRAS mutation, lasting 72 weeks, and 1 with UM with unknown mutation status, lasting 44 weeks). Another 9 patients had tumor decrease of at least 10% but did not meet criteria for PR. Several patients had prolonged SD on study suggesting possible clinical benefit.	Approximately 13% of patients had 1 or more dose reductions during their treatment due to AEs. Approximately 51% of patients experienced a dose delay during treatment, mostly due to AEs.
20	Homsi J et al., 2010 [67]	The median number of treatment cycles was 1 (range 1–7 cycles). 7 patients (32%) had SD with a median duration of 3 months (range: 3–7 months). The median OS was 9.8 months.	Neutropenia (23%) and musculoskeletal pain (10%) were the most common grade 3 and grade 4 toxicities.
21	Borden EC et al., 2011 [68]	Although a single patient had a sustained regression, overall IFN-b1a did not have clinical benefit (response rate <10%; median PFS 1.8 months).	Reversible drug-related severe (grade 3) AEs in 13/21 patients; anorexia and fatigue were mostly of mild or moderate severity.
22	Danielli R et al., 2012 [69]	No objective responses were observed; however, 2 patients had SD, a third patient had SD after initial PD. Median OS was 36 weeks (range 2–172 weeks).	No grade 3/4 AEs of non-immune origin were reported; 23% experienced grade 3 irAEs that resolved with steroid therapy.
23	Tarhini AA et al., 2011 [70]	Among 40 patients evaluable for efficacy, 7.5% had a confirmed PR, and 50% had PFS of ≥4 months. The 1 year survival rate is 56.4% (95% CI 43%–74%), *p* < 0.005. Median OS in this trial is 16.3 months, (95% CI 9.2 months–not reached).	Grade 3/4 toxicities included hypertension in 22% and proteinuria in 15%. AEs leading to treatment discontinuation included recurrent grade 3 proteinuria, grade 4 cerebrovascular ischemia, grade 3 left ventricular diastolic dysfunction and osteonecrosis of the mandibular bone.
24	Bhatia S et al., 2012 [71]	No confirmed objective responses occurred among the 24 evaluable patients (95% CI: 0–14%) and the study was terminated. The median PFS was 4 months (95% CI: 1–6 months) and the 6-month PFS was 29% (95% CI: 13%–48%). The median OS was 11 months (95% CI: 7–14 months).	29% experienced grade 4 AEs, all hematologic; 3 patients discontinued due to toxicity (myelosuppression or neuropathy); 75% required dose modifications due to toxicity.
25	Falchook GS et al., 2012 [72]	Among the 36 BRAF-mutant patients, 30 were BRAF-inhibitor naïve. Among these patients, 2 confirmed CRs and 8 confirmed PRs (RR = 33%); the median PFS was 5.7 months (95% CI, 4·0–7·4). Among the 6 BRAF-mutant patients who received prior BRAF inhibitor therapy, 1 unconfirmed PR was observed. Among 39 patients with BRAF wild-type melanoma, 4 PRs were observed (RR = 10%). Among the 16 patients with UM, 13% achieved a 24% tumor reduction. SD for ≥16 weeks was observed in 25%, including 2 who received treatment for >40 weeks.	The most common TEAEs were rash/dermatitis acneiform (80 out of 97; 82%) and diarrhea (n = 44; 45%), most of which were grade 2 or lower.
26	Ott PA et al., 2013 [73]	No objective responses were seen in any of the three patient cohorts. The best overall response was SD (31%); 71% had progressive disease at the 1 response assessment. In the phase II patient cohort, 23.5% patients had SD. The longest duration of SD in the cohort receiving the highest dose was 169 days. SDs >6 months occurred in patients with UM. The median duration of SD in the UM patients was 141 days (range: 57–337 days). The median TTP for the 29 evaluable patients was 57 days, whereas 113 days for the patients with UM.	No DLTs were seen in the first cohort (40 IU/m^2^). In cohort 2 (80 IU/m^2^), 1 grade 3 episode of arthralgia. No DLTs were observed in cohort 3 and enrollment onto the phase II part of the protocol was continued. MTD was not reached. Overall, the treatment was well tolerated; 6 grade 3 toxicities were observed in total.
27	Mahipal A et al., 2012 [74]	1 patient achieved a PR and 12 had a SD, with the duration of SD ranging from 2.1 to 29.2 months (median = 5.5 months). The patient who achieved a PR remained on treatment for 13 months. The median OS and PFS were 8.2 and 4.2 months, respectively; 3 patients had SD for more than 12 months with sunitinib after failing previous treatments.	The most common AEs were fatigue (90%), diarrhea (60%), hemorrhage (55%), anorexia (45%), hand-foot syndrome (25%), hypothyroidism (25%), and rash (25%); 11 patients required dose reduction due to grade 3 AEs.
28	Lane AM et al., 2009 [75]	Among IFN-treated patients, 34.7% developed metastasis compared with 27.5% in the control group; 31% developed metastasis after completing the 2-year course, and 13 died of the disease. The proportion of patients who died of metastatic disease was similar in the 2 groups: 30.6% in the IFN-group and 26.9% in the proton-therapy or enucleation only group. The 5-year melanoma-related death rates were 16.9% (95% confidence interval, 12.7%–22.4%) in the radiation or enucleation only group and 23.8% (95% confidence interval, 17.1%–32.6%) in the adjuvant therapy group. No differences were observed with longer follow-up.	Symptoms or onset of new illnesses (37.9%) and abnormal laboratory values (34.8%), were the most common reasons for discontinuing therapy. DLTs occurred in 28 patients and included thrombocytopenia (10.7%), elevated liver enzymes (42.9%), and thyroid function alterations (35.7%). All patients reported flu-like symptoms after the first few injections. Most AEs were mild to moderate in severity and resolved with continued IFN-therapy.
29	Hofmann UB et al., 2009 [76]	A total of 9 patients (75%) received imatinib for 8 weeks; 25% discontinued because of disease progression. No patient achieved an objective response; the best clinical response was a SD in 1 patient, which lasted for 52 weeks. Thus, the median PFS was not calculated. The median OS of all patients was 6.8 months. For the 8 patients who received imatinib as 1 line therapy, the median OS was 7.8 months. The 4 patients treated in a 2-line setting had a median OS of 4.9 months.	Abdominal pain and vomiting was the most common toxicity, resulting in a dose reduction in 2 cases (17%); 1 patient had facial edema. There was no significant hematologic toxicity.
30	Bedikian AY et al., 2008 [77]	1 CR, 2 PRs, and 5 SDs were observed in the 26 evaluable patients, resulting in a DCR of 31%. The median TTP was 1.9 months (95% CI: 1.8–2.2 months). The median survival was 9.6 months; (95% CI: 7.3–32 months) with 30% of the patients alive at 1 year. The median duration of SD was 4.2 months (range: 3.2–4.7 months).	Most of the side effects were grade 1–2 neurological, gastrointestinal, or constitutional. Nausea was the most common side effect. It occurred in about 75% of the patients and was usually mild. Neurologic AEs were constipation, hypoesthesia, anxiety, paresthesia, and peripheral neuropathy. The hematologic side effects were mild (mostly grade 1/2 neutropenia). None developed grade 3/4 thrombocytopenia.
31	Penel N et al., 2008 [95]	No objective response and only 1 SD with duration of 5 months were noted. No patient was found to be free of disease progression 6 months after the initiation of treatment. The overall survival was 10.8 months.	5 and 1 out of 13 enrolled patients experienced grade 3 and grade 4 toxicities, respectively. The most common severe AEs were abdominal pain.
32	Adjei AA et al., 2008 [78]	19 patients (33%) had SD at the end of cycle 2, and 9 patients (16%) had SD for ≥ 5 months; 1 patient with medullary thyroid cancer experienced SD for 19 cycles, whereas 1 patient with both UM and renal cell carcinoma had SD for 22 cycles.	Rash was the most frequent toxicity and DLT, occurring in 74% of all patients, and precluded dose escalation greater than 300 mg bid. Of the 43 episodes of skin rash, 34 were of maximum grade 1–2, and 9 were grade 3–4. Mild to moderate diarrhea was the principal gastrointestinal toxicity (56% of patients). Mild-moderate reversible ALT and AST elevation occurred in 14%; 14% experienced SAEs, including hypoxia, pneumonitis, bradycardia, renal insufficiency, and exfoliative dermatitis.
33	Schmittel A et al., 2006 [79]	7 confirmed SDs and 1 PR were observed in 24 patients treated with the GeT regimen, whereas no PR and only 3 SDs were observed in the T arm (*p* = 0.08). Median PFS was 3 months (95% CI 1.1–4.9) and 2 months (95% CI 1.7–2.3) in the GeT and T arm (*p* = 0.008, log-rank); 6 and 12 months PFS was 34.8% and 17.9% and 16.7% and 0% always favoring the GeT arm.	Grades 3 and 4 leukopenia only occurred in the GeT arm (17%; *p* = 0.001). 8% experienced a febrile neutropenia. Frequencies of anemia, nausea, vomiting, and infections were not significantly different in both treatment arms.
34	Richtig E et al., 2006 [80]	In 3 patients, therapy had to be withdrawn because of the appearance of metastases. Neither a univariate approach nor a multivariate approach could show a protective effect of interferon treatment on survival.	For 46% the initial dose had to be reduced due to leukopenia, thrombocytopenia, cardiac symptoms, elevated of liver function, or vertigo. In 5 patients, therapy had to be withdrawn because of serious side effects.
35	O’Neill PA et al., 2006 [81]	14 patients are evaluable for response. 4 patients completed all 6 cycles of chemotherapy. Of these, all 4 achieved SD after 3 cycles but 2 patients had progressed at re-assessment after cycle 6. The other 10 patients all had PD. Median PFS from the first cycle of chemotherapy was 12 weeks (2–26 weeks) and median OS was 30 weeks (2–64 weeks).	The treosulfan/dacarbazine combination was generally tolerated well. The major toxicities were hematological. Grade 1 or 2 thrombocytopenia was seen in 8 patients and grade 3 thrombocytopenia was seen in 3 patients. Grade 4 thrombocytopenia was also seen in 1 patient and Grade 4 neutropenia occurred in 1 patient. Non-hematological toxicity was generally mild with 2 patients experiencing grade 3 nausea and vomiting while 1 patient experienced grade 3 lethargy.
36	Schmittel A et al., 2005a [82]	In cohort 1 with a treosulfan dose of < or = 3000 mg/m2, no objective response was observed. Of the patients treated with > or = 3500 mg/m2 in cohort 2, 1 had PR (5%), 10 showed SD and 8 PD. An increased survival was observed in the second cohort with higher treosulfan doses, with median survival times of 6.0 versus 9.0 months (*p* = 0.03) in cohort 1 and 2, respectively, and a 1-year survival of 7.1% versus 47.3%, respectively.	Grade 3 thrombocytopenia was observed in 5/14 patients treated within cohort 1 and in 5/19 patients treated within cohort 2. Grade 3–4 leukopenia occurred in 2 patients in cohort 1 and 4 patients in cohort 2. 1 patient in cohort 2 had grade 3 anemia. No non-hematological AEs >grade 2 was observed.
37	Corrie PG et al., 2005 [83]	No objective CRs or PRs were documented. Best responses were 8% minor responses (both UM), 46% SD (10 cutaneous, 2 uveal) and 46% PD (10 cutaneous, 1 uveal). DCR was 54%. Median survival was 36 weeks (range 5–121). Median overall TTP was 14 weeks (range 3–74). Median survival and TTP for the UM patients were 53 weeks (range 20–103) and 27 weeks (range 7–38), respectively.	DLT was reached at 3.0 g m^2^ gemcitabine, when 2 of 6 patients experienced grade 3 myelosuppression. At the lower gemcitabine dose of 2.5 g m^2^, only 1 episode of grade 3 neutropenia occurred. Other common toxicities were nausea and vomiting, fatigue, skin rash and constipation.
38	Schmittel A et al., 2005b [84]	No objective response was observed; 7 patients (41%) had SD and 10 (59%) progressed. The median PFS of all 19 patients was 3.0 months (95% confidence interval (CI), 1.8–3.1); the median OS was 7.7 months (95% CI, 1.9–13.8). The 1-year survival was 31%.	Grade 3 and 4 leukopenia was observed in 9/19 patients. Grade 3 and 4 thrombocytopenia and leukopenia occurred in 8 and 9 patients, respectively. Grade 3 nausea, vomiting, and mucositis occurred in 1 patient each.
39	Schmidt-Hieber M et al., 2004 [85]	All 9 evaluable patients had progressive disease. 1 patient with progression experienced clinically significant symptom relief and therefore received 6 cycles. 2 patients died after the first cycle because of progressive disease.	Grade 3 and 4 toxicity consisted of anemia, thrombocytopenia, and leukocytopenia in 2, 1, and 2 patients, respectively. 3 patients showed grade 2 nausea, 1 patient grade 2 diarrhea and 1 patient grade 2 to 3 drug fever.
40	Keilholz U et al., 2004 [86]	For cases treated at dose levels 1/2, no objective responses were observed, whereas 2 patients (UM, renal cancer) on dose level 3 and 1 patient on dose level 4 (ovarian cancer) had a PR. Furthermore, we observed a stabilization of disease for more than 3 months in 15 patients with UM. A significant trend for improved OS with higher treosulfan doses was recorded.	Chemotherapy was generally well tolerated. Acute toxicity consisted of mild nausea in 9 patients. The predominant delayed toxicity was myelotoxicity. Grade 3 or 4 thrombocytopenia was observed in 3 and 1 patient, respectively. On dose levels 3 and 4, 1 and 2 patients, respectively, developed thrombocytopenia requiring a dose reduction. Grade 3 leukopenia was observed in 1 patient on dose level 3. Non-hematological side effects > grade 2 were alopecia and neutropenic fever.
41	Terheyden P et al., 2014 [87]	No patient achieved an objective response, 25% of patients (95% confidence interval, 8.6–49.1%) had stabilization of disease. The median time to progression for the patients achieving a SD was 187 days (range 182–316 days), with a prolonged median OS of 17 months compared with 7 months for the patients with PD. The median OS was worst in patients receiving treosulfan/gemcitabine as first-line therapy, i.e., 206.5 days (range 25–491 days).	The combination therapy of treosulfan/gemcitabine was well tolerated with no common toxicity criteria grade 2–4 non-hematological AEs.
42	Pföhler C et al., 2003 [88]	The analysis revealed 1 CR, 3 PR, and a SD in 8 cases. The objective response rate was 28.6%, the median OS was 61 weeks (95% confidence interval (CI) 54–133 weeks), the PFS was 28.5 weeks (95% CI 13–62 weeks), and the 1-year survival rate was 80%.	The drugs were well tolerated. The most common side effects were leukocytopenia and thrombocytopenia. 3 patients were withdrawn because of toxicity (thrombocytopenia grade 4). Grade 4 neutropenia occurred in only 1 patient.
43	Bedikian AY et al., 2003 [89]	No CR or PR were observed. SD was achieved in two patients. The median survival of the group was 6.7 months, with a range of 1–12.7 months. The median TTP was 1.84 months, with a range of 0.7–3.8 months.	Hematological toxicity was moderate; 3 patients developed grade 4 neutropenia, and 2 of these also developed grade 4 thrombocytopenia; 1 patient had grade 4 thrombocytopenia. Gastrointestinal side effects were the most common non-hematological toxic effects.
44	Kivelä T et al., 2003 [90]	None achieved an objective response (95% Confidence Interval (CI): 0–14), 8.3% remained stable, 20 showed progression. The median PFS was 1.9 months (95% CI: 1.8–3.4) and OS 10.6 months (95% CI: 6.9–16.4). OS improved with increasingly favorable pretreatment characteristics (median, 14.7 versus 6.9 versus 6.0 months for stages IVBa, IVBb, and IVBc, respectively; *p* = 0.018).	Grade 1–2 nausea, fever, flu-like syndrome, alopecia, hepatic toxicity, and neurotoxicity occurred in more than 30% of patients, and more than 10% experienced grade 3 alopecia and neurotoxicity.
45	Pyrhönen S et al., 2002 [91]	15% (95% confidence interval [CI] 0–38) obtained a partial objective response in hepatic and extrahepatic sites and 55% (95% CI 32–77) showed SD. The median PFS was 4 months (95% CI 2–10) and the median OS was 12 months (95% CI 8–22); 11 patients with Stage IVBa survived a median of 17 months (95% CI 4–37) whereas 10 patients with Stage IVBb survived a median of 11 months (95% CI 1–23).	Grade 1–2 malaise, fatigue, and fever were common. Almost 40% experienced grade 3–4 hematologic toxicity, and 35% developed grade 2–3 constipation. 2 treatment-associated deaths occurred (due to sepsis and myocardial infarction).
46	Becker JC et al., 2002 [92]	Only 1 patient (2%) achieved a CR and 6 (12.5%) a PR, for an ORR of 14.5% (95% confidence interval, 6.1 to 28.4%); 5 of these objective responses were observed in the cohort of patients receiving fotemustine HIA, while only 2 (1 CR and 1 PR) in the IV group experienced an objective response (21.7 vs. 8%). This difference did not translate into a significant benefit in OS, i.e., 369 and 349 days, respectively.	The most prominent side effect due to fotemustine was thrombocytopenia but never exceeded grade 3. A more prominent systemic toxicity of IV infusion was the more common occurrence of leukocytopenia. Thrombocytopenia was observed in 12 patients within the IV group in contrast to 4 patients in the HIA group (*p* = 0.028); 2 patients receiving intra-arterial fotemustine developed gastroenteric complications.
47	Ellerhorst JA et al., 2002 [93]	No CR or PR were observed. SD was achieved in 4 individuals (15%) for durations of 3, 4, 6, and 8 months; 2 of these patients had UM and 2 had cutaneous primaries. Disease progressed in spite of treatment in 22 individuals (85%).	17.9% developed grade 4 neutropenia and 7.1% grade 4 thrombocytopenia. 43% experienced grade 3 or 4 diarrhea and 18% grade 3 or 4 vomiting. Dehydration secondary to gastrointestinal toxicity lead to 4 hospitalizations. Myalgia and fatigue were also common, but were usually described as mild to moderate in intensity.
48	Mertens WC et al., 1996 [94]	1 PR was achieved for an ORR of 6% (95% CI 0–29) in a patient with UM metastatic to the liver, after 4 months of therapy, and lasted a further 8 months; 7 patients had SD (range 2–8 months), and lasting 3 months; 9 patients were found to have PD 6 weeks after initiation of treatment.	10 patients were able to escalate to 75 mg 3 times daily, but, of those, 3 required dose reductions because of toxicity. Of the 7 other patients, 5 could not escalate to a higher dose; 2 other patients required decrease in dosage of indomethacin.

ADRs = adverse drug reactions; AEs = adverse events; CP = carboplatinum/paclitaxel; CR = complete response; DCR = disease control rate; DoR = duration of response; DTL = dose-limiting toxicity; GeT = gemcitabine plus treosulfan; IrAEs = immune-related AEs; MFS = metastasis-free survival; MTD = maximum tolerated dose; ORR = overall response rate; OS = overall survival; QD = once daily; PFS = progression free survival; PD = progressive disease; PR = partial response; RR = response rate; SAEs = serious adverse events; SD = stable disease; T = treosulfan alone; TEAEs = treatment emergent adverse events; TTP = time to progression; UM = uveal melanoma.

**Table 7:** Ongoing clinical trials in patients with uveal melanoma.

(**a**) Local treatment or prevention of relapse and of metastatic disease or prevention of local complications in locally treated uveal melanoma.				
**N**	**Local treatment or Prevention of Relapse and of Metastatic Disease or Prevention of Local Complications in Locally Treated Uveal Melanoma**	**ID**	**Phase**	**Status**
1	Dexamethasone Implant for Retinal Detachment in Uveal Melanoma	NCT04082962	1	Recruiting
2	Crizotinib in High-Risk Uveal Melanoma Following Definitive Therapy	NCT02223819	2	Active, not recruiting
3	Stereotactic Body Radiation Therapy and Aflibercept in Treating Patients with Uveal Melanoma	NCT03712904	2	Recruiting
4	Adjuvant Sunitinib or Valproic Acid in High-Risk Patients with Uveal Melanoma	NCT02068586	2	Recruiting
5	Phase II Trial to Evaluate Safety and Efficacy of AU-011 Via Suprachoroidal Administration in Subjects with Primary Indeterminate Lesions and Small Choroidal Melanoma	NCT04417530	2	Recruiting
6	Neoadjuvant and Adjuvant Checkpoint Blockade	NCT02519322	2	Recruiting
7	Dendritic Cells Plus Autologous Tumor RNA in Uveal Melanoma	NCT01983748	3	Recruiting
8	Study in Subjects with Small Primary Choroidal Melanoma	NCT03052127	1/2	Active, not recruiting
9	Follow-up of Patients with Uveal Melanoma Adapted to the Risk of Relapse (SALOME)	NCT04424719	Not applicable	Recruiting
10	Influence of Oral Treatment with Citicoline for the Prevention of Radiation Optic Neuropathy in Patients Treated for Uveal Melanomas with Proton Beam Therapy	NCT01338389	Not applicable	Active, not recruiting
11	Endoresection of the Tumor Scar or Transpupillary Thermotherapy for the Treatment of Large Uveal Melanomas (Endoresection-Laser)	NCT02874040	Not applicable	Recruiting
(**b**) Metastatic or unresectable uveal melanoma.				
**N**	**Treatment of Metastatic or Unresectable Uveal Melanoma**	**ID**	**Phase**	**Status**
1	Intermittent Selumetinib for Uveal Melanoma	NCT02768766	1	Recruiting
2	A Phase I Study of LXS196 in Patients with Metastatic Uveal Melanoma.	NCT02601378	1	Active, not recruiting
3	Study of Immunotherapy Plus ADI-PEG 20 for the Treatment of Advanced Uveal Melanoma	NCT03922880	1	Active, not recruiting
4	Isolated Hepatic Perfusion in Combination with Ipilimumab and Nivolumab in Patients with Uveal Melanoma Metastases (SCANDIUM II)	NCT04463368	1	Recruiting
5	Autologous CD8+ SLC45A2-Specific T Lymphocytes with Cyclophosphamide, Aldesleukin, and Ipilimumab in Treating Patients with Metastatic Uveal Melanoma	NCT03068624	1	Active, not recruiting
6	IKKb-matured, RNA-loaded Dendritic Cells for Metastasized Uveal Melanoma	NCT04335890	1	Recruiting
7	A Study of RO7293583 in Participants with Unresectable Metastatic Tyrosinase Related Protein 1 (TYRP1)-Positive Melanomas	NCT04551352	1	Recruiting
8	C7R-GD2.CART Cells for Patients with Relapsed or Refractory Neuroblastoma and Other GD2 Positive Cancers (GAIL-N)	NCT03635632	1	Recruiting
9	Study of Safety and Tolerability of BCA101 Alone and in Combination with Pembrolizumab in Patients with EGFR-driven Advanced Solid Tumors	NCT04429542	1	Recruiting
10	A Safety and Tolerability Study of INCAGN02390 in Select Advanced Malignancies	NCT03652077	1	Active, not recruiting
11	Modified Virus VSV-IFNbetaTYRP1 in Treating Patients with Stage III-IV Melanoma	NCT03865212	1	Recruiting
12	Intravenous and Intrathecal Nivolumab in Treating Patients with Leptomeningeal Disease	NCT03025256	1	Recruiting
13	A Study to Assess PV-10 Chemoablation of Cancer of the Liver	NCT00986661	1	Recruiting
14	IN10018 Monotherapy and Combination Therapy for Metastatic Melanoma	NCT04109456	1	Recruiting
15	A Phase II Study of BVD-523 in Metastatic Uveal Melanoma	NCT03417739	2	Active, not recruiting
16	Nivolumab Plus Relatlimab in Patients with Metastatic Uveal Melanoma	NCT04552223	2	Recruiting
17	Transarterial Radioembolization in Comparison to Transarterial Chemoembolization in Uveal Melanoma Liver Metastasis (SirTac)	NCT02936388	2	Recruiting
18	Safety and Efficacy of IMCgp100 Versus Investigator Choice in Advanced Uveal Melanoma	NCT03070392	2	Active, not recruiting
19	Trial of Nivolumab in Combination with Ipilimumab in Subjects with Previously Untreated Metastatic Uveal Melanoma (GEM1402)	NCT02626962	2	Active, not recruiting
20	SIR-Spheres^®®^ 90Y Microspheres Treatment of Uveal Melanoma Metastasized to Liver	NCT01473004	2	Active, not recruiting
21	Nivolumab and Ipilimumab in Treating Patients with Metastatic Uveal Melanoma	NCT01585194	2	Active, not recruiting
22	Efficacy Study of Pembrolizumab with Entinostat to Treat Metastatic Melanoma of the Eye (PEMDAC)	NCT02697630	2	Active, not recruiting
23	Adoptive Transfer of Tumor Infiltrating Lymphocytes for Metastatic Uveal Melanoma	NCT03467516	2	Recruiting
24	Ipilimumab and Nivolumab with Immunoembolization in Treating Participants with Metastatic Uveal Melanoma in the Liver	NCT03472586	2	Recruiting
25	A Trial of Niraparib in BAP1 and Other DNA Damage Response (DDR) Deficient Neoplasms (UF-STO-ETI-001)	NCT03207347	2	Recruiting
26	Cabozantinib-S-Malate Compared with Temozolomide or Dacarbazine in Treating Patients with Metastatic Melanoma of the Eye That Cannot Be Removed by Surgery	NCT01835145	2	Active, not recruiting
27	Iodine I 131 Monoclonal Antibody 3F8 in Treating Patients with Central Nervous System Cancer or Leptomeningeal Cancer	NCT00445965	2	Active, not recruiting
28	The Scandinavian Randomized Controlled Trial of Isolated Hepatic Perfusion for Uveal Melanoma Liver Metastases (SCANDIUM)	NCT01785316	3	Recruiting
29	Percutaneous Hepatic Perfusion in Patients with Hepatic-dominant Ocular Melanoma (FOCUS)	NCT02678572	3	Active, not recruiting
30	Study of PAC-1 and Entrectinib for Patients with Metastatic Uveal Melanoma	NCT04589832	1/2	Not yet recruiting
31	A Study of the Intra-Patient Escalation Dosing Regimen with IMCgp100 in Patients with Advanced Uveal Melanoma	NCT02570308	1/2	Active, not recruiting
32	Yttrium90, Ipilimumab, & Nivolumab for Uveal Melanoma with Liver Metastases	NCT02913417	1/2	Recruiting
33	PHP and Immunotherapy in Metastasized UM (CHOPIN)	NCT04283890	1/2	Recruiting
34	Safety & Activity of Controllable PRAME-TCR Therapy in Previously Treated AML/MDS or Metastatic Uveal Melanoma	NCT02743611	1/2	Active, not recruiting
35	Study of IDE196 in Patients with Solid Tumors Harboring GNAQ/11 Mutations or PRKC Fusions	NCT03947385	1/2	Recruiting
36	A Study of PLX2853 in Advanced Malignancies.	NCT03297424	1/2	Recruiting
37	Hypofractionated Stereotactic Linear Accelerator Radiotherapy of Uveal Melanoma	NCT00872391	Not applicable	Recruiting
38	Communicating with Patients on Cancer Resistance to Treatment: the Development of a Communication Tool (HECTOR)	NCT04118062	Not applicable	Not yet recruiting

## Data Availability

Publicly available datasets were analyzed in this study. These data can be found here: https://pubmed.ncbi.nlm.nih.gov/, (accessed on 1 February 2021); https://clinicaltrials.gov/, (accessed on 15 February 2021).
